# Circular RNAs in Cardiovascular Diseases: Regulation and Therapeutic Applications

**DOI:** 10.34133/research.0038

**Published:** 2023-01-13

**Authors:** Lijun Wang, Gui-e Xu, Michail Spanos, Guoping Li, Zhiyong Lei, Joost P. G. Sluijter, Junjie Xiao

**Affiliations:** ^1^ Cardiac Regeneration and Ageing Lab, Institute of Geriatrics (Shanghai University), Affiliated Nantong Hospital of Shanghai University (The Sixth People’s Hospital of Nantong), School of Medicine, Shanghai University, Nantong 226011, China.; ^2^ Institute of Cardiovascular Sciences, Shanghai Engineering Research Center of Organ Repair, School of Life Science, Shanghai University, Shanghai 200444, China.; ^3^ Cardiovascular Division of the Massachusetts General Hospital and Harvard Medical School, Boston, MA 02114, USA.; ^4^ Department of Cardiology, Laboratory of Experimental Cardiology, University Medical Center Utrecht, Utrecht 3508GA, The Netherlands.; ^5^ CDL Research, University Medical Center Utrecht, Utrecht 3584CX, The Netherlands.; ^6^ Regenerative Medicine Center, Circulatory Health Laboratory, University Medical Center Utrecht, University Utrecht, Utrecht 3508GA, The Netherlands.

## Abstract

Cardiovascular disease is one of the leading causes of mortality worldwide. Recent studies have shown that circular RNAs (circRNAs) have emerged as important players in the prevention and treatment of cardiovascular diseases. circRNAs are a class of endogenous noncoding RNAs that are generated by back-splicing and are involved in many pathophysiological processes. In this review, we outline the current research progress on the regulatory roles of circRNAs in cardiovascular diseases. Further, new technologies and methods available for identifying, validating, synthesizing, and analyzing circRNAs, as well as their applications in therapeutics, are highlighted here. Moreover, we summarize the increasing insights into the potential use of circRNAs as circulating diagnostic and prognostic biomarkers. Finally, we discuss the prospects and challenges of circRNA therapeutic applications for cardiovascular disease therapy, with a particular focus on developing circRNA synthesis and engineering delivery systems.

## Introduction

Circular RNAs (circRNAs) are a class of covalently closed RNA sequences without 5′- cap and 3′-polyadenylate [poly(A)], most of which do not have protein-coding abilities [1]. circRNAs were first recognized as by-products of RNA mis-splicing, and, thus, their functions were long overlooked. Until recent years, with the advances in high-throughput sequencing technology, more-and-more circRNAs have been identified and investigated for their underlying regulatory functions [2–5]. Compared with linear RNA, circRNA is more stable and resistant to RNA exonuclease degradation. In eukaryotic cells, circRNAs are abundantly expressed with high evolutionary conservation and tissue specificity [6–10]. A deeper understanding of circRNAs can provide insight into disease pathogenesis, potential treatments, and their use for monitoring disease prognosis.

**Fig. 1.**
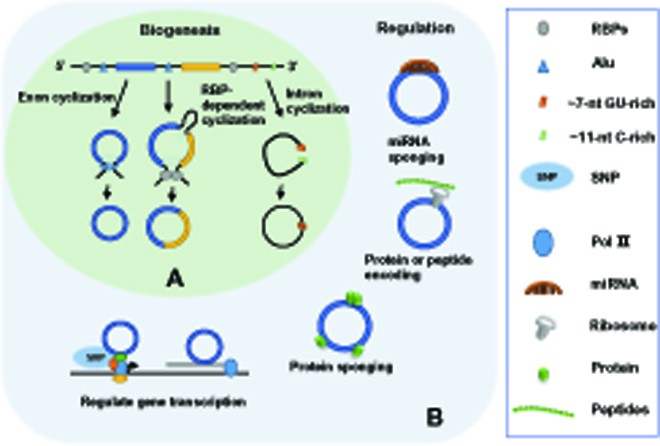
Overview of circRNA biogenesis and regulation. (A) Three main biogenesis mechanisms of circRNAs: exon cyclization, intron cyclization, and RBP-dependent cyclization. (B) circRNA exerts its regulatory functions by miRNA sponging protein sponging, gene transcription regulation, or peptide (or protein) encodings. SNP, small nuclear ribonucleoproteins; nt, nucleotide; Pol II, polymerase II.

On the basis of their sequence composition, circRNAs can be classified as exonic circRNAs, intronic circRNAs, and exon–intron circRNAs [11]. circRNAs are mainly formed by the back-splicing of precursor mRNAs. There are 3 major biogenesis mechanisms associated with circRNAs: exon cyclization, intron cyclization, and RNA binding protein (RBP)-dependent cyclization (Fig. 1) [12–14]. Among them, exon circularization can be further divided into lariat-driven cyclization and direct back-splicing mode, according to the sequence of splicing events [12,13]. If canonical splicing occurs first, then a lasso structure containing skipped exons will be generated first, whereafter the exons are spliced in the lasso to form circRNAs, a pattern called lasso mode [12]. Whereas if back-splicing occurs first, circRNA along with an exon–intron–exon intermediate will be directly generated, and this mode is called direct back-splicing mode [13]. Alternatively, the flanking introns at both ends of the exons contain multiple pairs of reverse complementary sequences, which can promote the pairing of the intron sequences, thereby bringing the downstream splice donor and the upstream splice acceptor closer together to form a circRNA [15–17]. Another way that promotes looping is RBP binds directly to upstream and downstream introns flanking sequences of exons [14,18–20]. Moreover, some of the intronic circRNAs are self-cyclized by 2′-5′ phospholipid connections and located in the nucleus [21]. Finally, in addition to intron sequences and spliceosomes, RNA polymerase II can also interact with RNA sequences and, thus, can be involved in forming circRNAs that contain both exons and introns [22].

Given the diversity of circRNAs and their many individual distributions, circRNAs are expected to play many important cellular and molecular roles (Fig. 1). circRNAs were first identified as microRNA (miR) sponges [3,23]. For example, ciRS-7, one of the first well-characterized circRNAs, contains ~70 miR-7 binding sites and thereby inhibiting its activity [23]. However, increasing numbers of studies have realized that the competitive endogenous RNA inhibitory mechanism optimally occurs when the miRNA and target are close to equimolar concentrations, but most of the circRNAs do not contain such a unique and sufficient number of miRNA binding sites [24]. At the same time, more regulatory mechanisms for circRNAs are being discovered. circRNAs can exert biological functions by binding to RBP, modulating gene transcription, and regulating the alternative splicing of their parental genes [25,26]. Interestingly, similar to linear long noncoding RNAs (lncRNAs), some circRNAs may also have coding potential and can encode small peptides or proteins that exert regulatory function [27–31]. Nevertheless, so far, the biogenesis and regulatory functions of circRNAs have not been fully elucidated, and further studies are needed.

Cardiovascular disease (CVD) is one of the leading causes of human death imposing a heavy economic burden on medical systems worldwide [32,33]. Large numbers of scientific and clinical research are devoted to the diagnosis, treatment, and prognosis of CVDs. However, effective treatments and therapeutics for CVD are largely lacking. Because of the deeper understanding of the RNA world, RNA-based therapeutic strategies have gained wide attention in recent years. circRNAs are involved in various cellular processes and have been associated with the occurrence and development of many human diseases [34–36]. The unique structure and properties of circRNAs make them ideal candidates for biomarkers and have broad development prospects in disease diagnosis and treatment [9,37]. Regarding the cardiovascular system, studies have shown that circRNAs are important mediators of CVDs including ischemic heart disease, cardiac fibrosis, atherosclerosis, cardiomyopathy, and cardiac senescence [38–41]. This review outlines the current status of circRNA research in CVDs, emphasizing technical and methodological progress as well as its application in CVD treatment. We will also discuss the prospects and challenges of circRNA therapeutic applications for future CVD therapy.

## Techniques and Databases Used for circRNAs in the Cardiovascular System

**Fig. 2.**
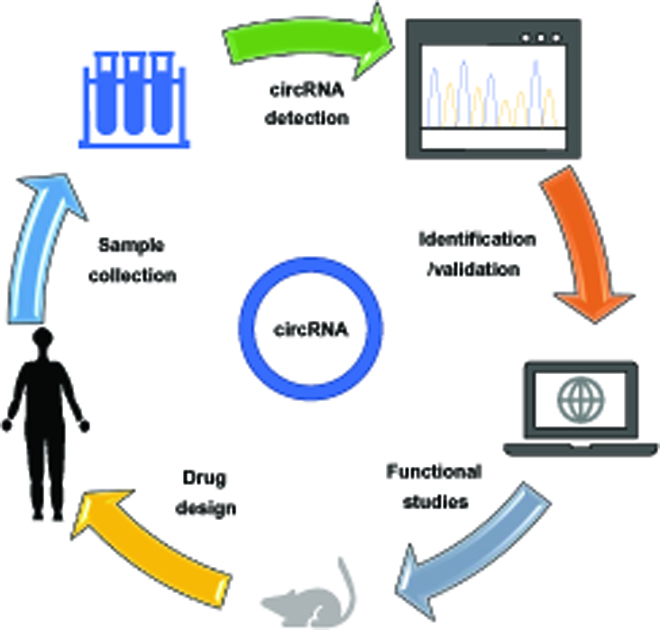
Workflow of circRNA study in cardiovascular research. Samples were collected from human or animal tissues/body fluids, and circRNAs are identified. Next, functional studies are conducted to confirm the mechanism of action of candidate circRNAs. Finally, drugs or biomarkers are developed for medical research and clinical applications.

**Fig. 3.**
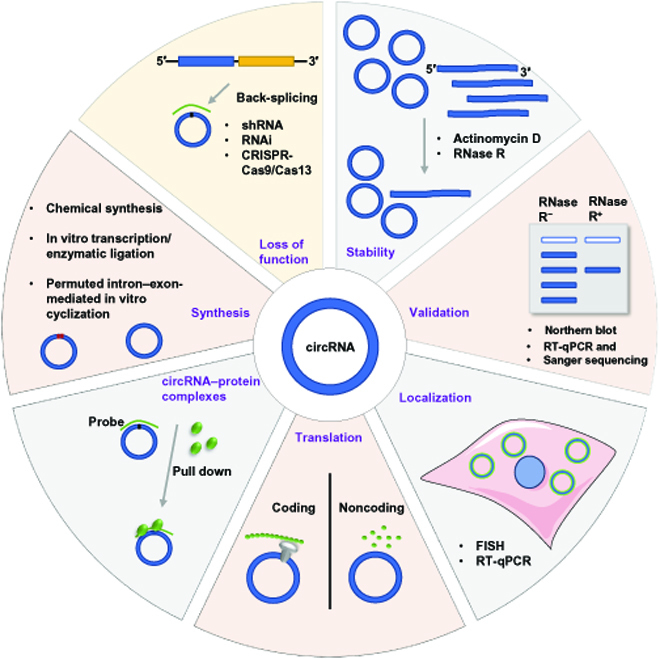
General overview of the process of circRNA functional characterization in cardiovascular system. Methodologies that are usually implemented in circRNA characterization (validation, stability, and localization), functional determination (loss of functional assay, translation, synthesis, and circRNA interaction protein identification), FISH (fluorescence in situ hybridization), and RNA FISH. shRNA, short hairpin RNA; RNAi, RNA interference.

The process that leads from studying circRNA through basic and clinical research to developing novel circRNA-based theranostics is a multistep and lengthy one (Fig. 2). We will not discuss basic knowledge and practice principles in this section, as they have been well organized and discussed in recent reviews [42–44]. Instead, here, we will focus on recent developments in technologies, methods, and databases pertaining to the identification, validation, and functional studies of circRNAs in the cardiovascular system (Fig. 3).

### Progression in circRNA detection and validation approaches

Because of lack of poly(A) tails and their low abundance in cells, circRNAs have long been ignored. With advanced deep sequencing technologies and enrichment methodologies, circRNAs are identified and annotated by mapping back-splicing junction (BSJ) sites in high-throughput sequencing data (Table 1). Ribosomal RNA (rRNA) depletion and ribonuclease (RNase) R treatment are currently 2 commonly used experimental approaches in circRNA library preparation. In most cases, RNase R treatment preferentially degrades linear RNAs and is often used for circRNA enrichment. However, some linear RNAs are naturally resistant to RNase R either because of high G-quadruplex content or tightly structured 3′ end, and, thus, replacing K^+^ to Li^+^ in the RNase R reaction buffer is important for the efficient degradation of such linear RNAs [45]. Besides, although RNase R treatment is sufficient to enrich enough circRNA and contribute to the discovery of circRNAs, profiling circRNA expression in total RNAs without RNase R treatment is equally important to determine the signal of circRNA coexpression with other kinds of RNAs. After library preparation and deep sequencing, a specific algorithm is required to align the RNA sequences and map them to the BSJ locus for circRNA identification. Recently, a new algorithm called CIRI-long using nanopore technology for full-length circRNA sequencing has been developed [46]. Although, currently, most of the circRNAs are identified via BSJ mapping in cardiovascular studies, it is worth mentioning here that circRNA annotated via the targeting BSJ site lacks the internal sequence information, especially for those circRNAs composed of multiple exons. Long-read deep sequencing, such as CIRI-long, provides the opportunity to distinguish circRNAs with the same BSJ site but different exon compositions [47]. Moreover, because of the low abundance of circRNAs, more than 2 algorithms should be applied for identification. This will minimize the number of false positives and obtain reliable annotations for further experimental verification [48].

**Table 1.** Techniques used in circRNA cardiovascular research.

**Techniques**	**Research purpose**
rRNA-depletion RNA sequencing	circRNA detection
Poly(A)-depletion RNA sequencing	circRNA detection
RNase R-treatment RNA sequencing	circRNA detection
RT-PCR/Sanger sequencing for BSJ site	circRNA validation
Northern blot	circRNA validation
RNA FISH	circRNA subcellular location
Nucleus and cytoplasmic separation, RT-qPCR	circRNA subcellular location
Short interfering RNAs, short hairpin RNAs	circRNA inhibition
CRISPR-Cas9-based genome editing	circRNA inhibition
CRISPR-Cas13d-based RNA depletion	circRNA inhibition
Overexpression in vivo by plasmids or virus delivery	circRNA overexpression
In-vitro-synthesized circRNA	circRNA overexpression

Experimental validation of circRNAs is essential before specific characterization and functional assays can be conducted (Table 1). Both polymerase chain reaction (PCR)-based techniques and Northern blotting can be applied to validate circRNAs in cardiovascular research. Reverse transcription PCR (RT-PCR) is the most used method for circRNAs’ first-step validation. Usually, primers designed to cross the BSJ site of circRNAs, agarose gel, and Sanger sequencing are applied to the PCR products to confirm the precise cyclization sites. It should be noted that because of their relatively low abundance in the circulating system, circRNAs are detected mostly using PCR-based methods. In addition to PCR-based validation, the Northern blot is also a useful method to verify the existence of circRNA and distinguish it from its linear counterpart mRNAs by differential migration rate on a gel. To perform a Northern blot assay for circRNA, an abundance of circRNA should be achieved, otherwise an RNase R enrichment assay should be performed prior, to ensure that BSJ-targeting probes are sufficiently hybridized to circRNAs.

### Methodologies for circRNA functional determination

The subcellular localization of cellular circRNAs is important for determining their potential regulatory role, as it has been with other types of RNAs. Most of the exon-derived circRNAs are localized in the cytoplasm, while intron-containing circRNAs are mostly in the nucleus. RT quantitative PCR (RT-qPCR) and RNA fluorescence in situ hybridization (FISH) are the most widely used approaches to determining the subcellular localization of circRNAs (Table 1). RT-qPCR is generally popular for its simple operation and easy detection. However, this methods’ detection accuracy depends on the purity of the nucleus and cytoplasmic extraction. RNA FISH specificity for circRNAs is limited to the choice of probe specifically designed to cross the BSJ site and hybridize with the circRNAs. Nonetheless, it is critical to perform both RT-qPCR and RNA FISH to minimize false-positive signal caused by other organelle-localized RNAs and their counterpart mRNAs. Therefore, cross-validation of more than 2 methods is often required to illustrate the subcellular localization of circRNAs. In addition, RNA FISH can provide the suborganelle localization of circRNAs in the cytoplasm, such as mitochondria-localized circRNA [49]. Finally, given the high abundance of mitochondria in cardiomyocytes, studying the mitochondria-derived circRNAs or circRNAs that are localized in the mitochondria is critical to better understand the regulatory role of cardiac circRNAs.

Once the existence and subcellular location of circRNAs have been verified, the next logical step is exploring the biological function of the identified circRNA. Gain- and loss-of-function studies are commonly used approaches to determining the functional phenotypes of circRNAs in cells and animals (Table 1). Many strategies have been employed to inhibit circRNAs. Short interfering RNAs or short hairpin RNAs that target the BSJ site of circRNAs are the most useful strategy for specific knockdown of circRNAs in cardiovascular studies. Importantly, when using this method to suppress circRNA expression, precautions should be taken not to co-degrade the linear mRNA along with circRNA. As a rule of thumb, the linear mRNA levels should be measured simultaneously with circRNA. An additional circRNA depletion strategy is CRISPR-Cas9-based genome editing. In the scenario that a circRNA is derived from a locus that does not contain annotated genes, as in the case of Cdr1as, guide RNAs can be designed to directly ablate the genome locus, causing complete silencing and, thus, generating a Cdr1as knockout mouse [50]. However, most circRNAs are derived from exon regions and have marked sequence overlap with their linear counterparts. Using CRISPR-Cas9 technology, it is possible to delete the Alu elements, complementary intron sequences, or binding sites that flank circRNAs to promote their cyclization [51]. In addition, new technologies are being developed with advances in the field of circRNA. To name a few, base editors that combine nucleobase deaminases in a circRNA knockout strategy have been developed [52]. In addition, using CRISPR-Cas13d, where guide RNAs are designed against circRNA BSJ sites, is another effective method for silencing circRNAs [53,54]. The use of these novel technologies is expected to revolutionize cardiovascular research in the near future. In summary, it is imperative that all approaches used for inhibiting circRNAs take into account the potential effects on their cognate linear mRNAs and other back-spliced circRNAs derived from the same genomic location or host gene. In this sense, it is crucial that there are enough control assays to minimize any possibility that observed phenotypes are the consequence of unwanted target inhibition, e.g., linear mRNAs, other circRNAs, or other off-target effects. It is important to keep in mind that, as with any study of this kind, off-target information must be obtained and taken into account before circRNA is considered for clinical use in CVD.

circRNA overexpression can also be achieved by several approaches. Currently, the most used method in cardiovascular research is generating a circRNA overexpression construct and then makes use of either plasmids to transfect cells or adeno/lentivirus delivery. In general, the overexpression vector can be constructed by inserting the cyclized sequences flanking with native intron sequences that are sufficient for cyclization [23]. The use of modified artificial cyclization elements enabled efficient and precise cyclization of several circRNAs through overexpression vectors [16]. As a result of advances in circRNA research, new technologies to overexpress circRNA have been developed. The twister ribozyme expression system, also named Tornado (“Twister-optimized RNA for durable overexpression”), is designed to generate circRNA in vivo on the basis of ribozymes’ autocatalytic cleavage activity and then ligated by RNA ligase RtcB [55]. Moreover, with the advancement of RNA synthesis technologies, several circRNA synthesis strategies have been developed by in vitro cyclization and subsequently direct transfection of purified circRNAs into cells. This strategy is discussed in detail in the next section. Research in CVD will undoubtedly benefit from all the strategies discussed above, as well as the development of new technologies in the future.

### Methodologies for circRNAs synthesis in vitro

**Fig. 4.**
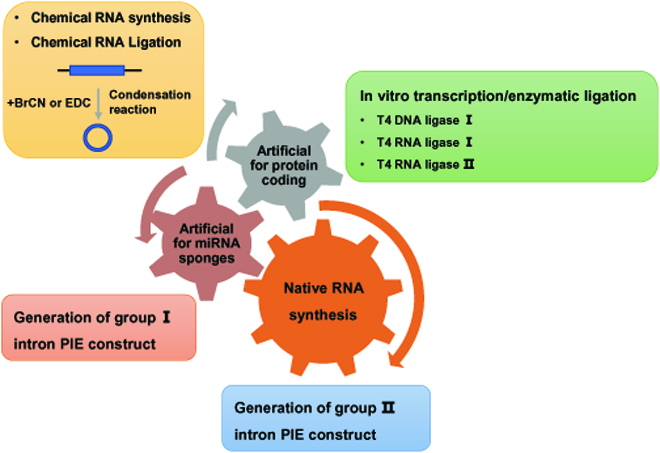
Strategies of engineered circRNA design. Four synthetic strategies (chemical synthesis, IVT/enzymatic ligation, group I intron PIE-mediated in vitro cyclization, and group II intron PIE-mediated in vitro cyclization) used for circRNA synthesis in vitro*.* Engineered circRNAs can be categorized into engineer-designed synthetic circRNAs (native RNA synthesis) and engineer-designed artificial circRNA. Engineered artificial circRNAs are designed to act as miRNA sponges or encode proteins. BrCN, cyanogen bromide; EDC, 1-ethyl-3-(3-dimethylaminopropyl) carbodiimide.

Synthesis of circRNA in vitro and the provision of RNA-based therapies for CVDs will undoubtedly have a major impact on health care in the future (Fig. 4). Normally, 2 types of circRNAs can be synthesized: native circRNAs with endogenous sequences and engineer-designed circRNAs. circRNAs are more stable than linear transcripts, and some circRNAs can encode for a protein/peptide via an internal ribosome entry site-driven or RNA *N*^6^-methyladenosine (m^6^A) methylation-driven protein translation. circRNA translation and its clinical significance have been well described in a recent review; therefore, we will not elaborate here [27]. Engineer-designed artificial circRNA can be designed to take advantage of aforementioned properties. Among engineered artificial circRNAs, those that encode a protein or act as miRNA sponges are the most explored. Currently, chemical synthesis is one of the most commonly used synthesis strategies available. Similar to linear RNAs, the molecule sizes of circRNAs are limited to shorter length sequences (usually less than 80 nucleotides) when chemical synthesis is applied. At present, there are several commonly used methods for preparing circRNAs for in vitro synthesis. More commonly, a linear form of circRNA is synthesized using in vitro transcription (IVT) as the first step, followed by enzymatic ligation by T4 bacteriophage DNA or RNA ligases [56–58]. T4 DNA ligase requires a 10- to 20-nucleotide DNA splint that can hybridize to the 5′ and 3′ end of linear RNAs [56]. T4 RNA ligases can also be applied to ligation IVT RNA substrate. RNA ligase I preferably ligates linear RNA so that the 5′ and 3′ termini are in close proximity, and then RNA ligase II would efficiently join the nicks in double-stranded RNAs. However, all the ligases mentioned above unavoidably generate intermolecular ligation by-products with circRNAs. For that reason, further purification procedures, including polyacrylamide gel electrophoresis, agarose gel separation, or high-performance liquid chromatography, should be explored to separate the circRNA products from intermolecular by-products, the remaining linear products, and other components introduced during the experiment. Moreover, it should be mentioned that the low enzyme ligation efficiency is another limiting factor to large-scale production applications of circRNAs.

Alternatively to enzymatic ligation by T4 ligases, using ribozymes sequences is another strategy to generate circRNAs. Specifically, group I and II introns have been applied to construct circRNAs in in vitro cyclization systems, also known as the permuted intron–exon (PIE) method [57,59,60]. This approach is generally based on autocatalytic activity from naturally existing intron sequences derived from bacteria and nonmetazoan eukaryotes. For group I intron, a backbone containing the intron sequences from the T4 bacteriophage thymidylate synthase (*td*) gene and *Anabaena* pre-tRNAs are most commonly used for circRNA circularization [57–59,61]. As to group II introns, it has been reported that a permuted group II intron from the yeast mitochondrial genome can be successfully used to generate circRNA in vitro [62,63]. In general, PIE methods result in higher circularization efficiency than enzymatic ligation using T4 ligases, but there are still some limitations. Group I intron cyclization requires retention of an extra spacer and exhibits a different junction site from the natural products. Moreover, while currently used group I introns can achieve precise cyclization at the junction sites, the underlying splicing mechanism is still not clear. Further efforts to describe the exact group I and II intron splicing mechanism and improve the circularization efficiency of circRNAs should be of priority. This will enable us to optimize the PIE system and extend its applications.

### Databases for circRNAs in cardiovascular research

As more and more circRNAs are identified, new databases are being developed to provide various types of information related to circRNAs (Table 2). CircBase is the first established circRNA database, which unified circRNA sequence datasets, and allows the direct download of circRNA sequences and the Python scripts needed to discover new circRNAs [64]. Databases, such as circBank, circCpedia v2, circRNADb, circAtlas 2.0, circSC, ExoRBase 2.0, and circFunBase, are also developed to annotate circRNAs. These databases often collected circRNA information including circRNA-spliced sequences, expression features, and genomic locations [65–71]. In addition to basic information about circRNAs, databases often have distinctive characteristics, which are summarized in Table 2. Aside from the identification and annotation of circRNAs, the StarBase v2.0, DeepBase v3.0, CircInteractome, CircNet 2.0, and TRCirc databases have also been established to collect and profile the RNA–RNA and protein–RNA interaction networks [72–76]. Moreover, as a result of the growing number of circRNA studies that have demonstrated that circRNA regulation plays an important role in CVD, some databases provide comprehensive information about the correlation between circRNA expression and CVD. Examples of such databases include Circ2Disease, lncRNADisease v2.0, and the Circad database [77–79]. While there are some issues that limit database development, including differences in nomenclature systems and the absence of full-length sequences of circRNAs, the advancement of database construction and algorithm optimization on circRNAs will undoubtedly contribute to the advancement of cardiovascular research in the future.

**Table 2.** Databases used in circRNA cardiovascular research.

**Name**	**Species**	**Key information**	**Latest update**	**URL**
**circRNA identification and annotation**				
CircBank	Human	Established a new nomenclature system based on the circRNAs’ host genes, provided the information of circRNA related to evolutionary conservation, m^6^A methylation, prediction of miRNA binding sites, circRNA coding potential, and point mutations	2018 [65]	http://www.circbank.cn/
CircBase	Human, mouse, worm	Unified circRNA sequence datasets, allows the direct download of circRNA sequences and the Python scripts used to discover new circRNAs	2017 [64]	http://circbase.org/
CircCpedia v2	Human, mouse, rat, zebrafish, fly, worm	Collected circRNA data from 180 RNA sequence datasets, provided computational tools to compare circRNA expression among samples and the conservation analysis between human and mouse	2018 [66]	http://yang-laboratory.com/circpedia/
CircRNADb	Human	Unified the identified circRNAs and their functions, with a special focus on the potential encoding possibility and gene expression regulation	2016 [67]	http://reprod.njmu.edu.cn/circrnadb
CircAtlas 2.0	Human, *Macaca*, mouse, rat, pig, chicken	Unified circRNAs in vertebrates, provided circRNAs’ expression and functional profile, related to the conservation analysis, open reading frames and internal ribosomal entry sites prediction, RBP and miRNA binding, as well as association with diseases	2020 [68]	http://circatlas.biols.ac.cn/
circSC	Human, mouse	Integrated the full-length single-cell RNA sequencing circRNA datasets	2022 [69]	http://159.226.67.237/zhao/Database/circSC/index.html
CIRI-hub	Human	A visual analytic platform for circRNAs in cancer to generate high-quality figures for analyzed data	2021	http://159.226.67.237/zhao/Database/CIRIhub/index.html
ExoRBase 2.0	Human	RNAs derived from extracellular vesicles in human body fluids	2021 [70]	http://www.exorbase.org/
CircFunBase	Human, mouse, *Solanum lycopersicum*, etc.	Documented both circRNAs experimentally validated and their computationally predicted biological functions, provided visualized circRNA–miRNA interaction networks	2019 [71]	http://bis.zju.edu.cn/CircFunBase/index.php
**circRNA interaction network**				
StarBase v2.0 (ENCORI)	Human, mouse, worm, fungi, nematode, insect, *Amborellales*, Cnidaria, eudicots, monocots, etc.	Profiled the RNA–RNA and protein–RNA interaction networks from cross-linking immunoprecipitation-sequencing and high-throughput sequencing data datasets	2022 [72]	http://starbase.sysu.edu.cn/
DeepBase v3.0	Human, mouse, *Gallus gallus*, *Pan troglodytes*, *Gorilla gorilla gorilla*, *Macaca mulatta*, rat, *Bos taurus*, *Monodelphis domestica*, *Ornithorhynchus anatinus*, *Xenopus tropicalis*, *Danio rerio*, *Caenorhabditis elegans*	Provided the comprehensive expression profile of small RNAs and lncRNAs, and the extracellular patterns of miRNAs, lncRNAs, and circRNAs were also integrated into this database	2020 [73]	http://rna.sysu.edu.cn/deepbase3/index.html
CircInteractome	Human	Provide information about circRNAs and their interacting map with RBPs and miRNAs	2020 [74]	http://circinteractome.nia.nih.gov
CircNet 2.0	Human	Profiled the circRNA–miRNA–gene regulatory network both from experimentally verified miRNA targets and from miRNA prediction tools of PITA, miRanda, and TargetScan	2021 [75]	https://awi.cuhk.edu.cn/~CircNet
TRCirc	Human	Provided information about circRNA transcriptional regulation, the methylation level, H3K27ac, and super-enhancers correlated with circRNA	2018 [76]	http://www.licpathway.net/TRCirc
**circRNA expression and CVDs**				
circ2Disease	Human, mouse, rat	Curated the comprehensive associations between circRNAs and various human diseases, integrated experimentally verified miRNAs, and miRNA targets	2018 [77]	http://bioinformatics.zju.edu.cn/Circ2Disease/index.html
Circad	Human, mouse, rat	Provided disease-associated circRNA information	2020 [79]	http://clingen.igib.res.in/circad/
lncRNADisease v2.0	Human	Integrated ncRNA disease association data	2019 [78]	http://www.rnanut.net/lncrnadisease/

## The Role of circRNAs in CVDs

**Fig. 5.**
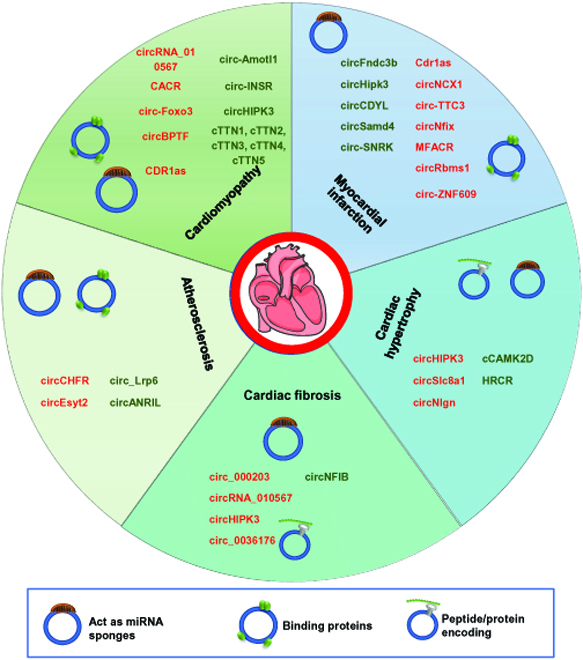
Summary of circRNAs in CVDs. The regulation of circRNAs involved in cardiac fibrosis, MI, atherosclerosis, cardiomyopathy, and cardiac hypertrophy. Green, downregulated; red, upregulated.

circRNAs have been found to be involved in the development of various human diseases. Here, we focus on the emerging regulatory and mechanistic roles of circRNA in CVDs, including cardiac fibrosis, myocardial infarction (MI), atherosclerosis, cardiomyopathy, and cardiac hypertrophy (Fig. 5 and Table 3).

**Table 3.** Summary of circRNAs in CVDs.

**circRNAs**	**Disease**	**Mechanism**	**Host gene**	**Expression**	**Source**	**References**
circFndc3b	MI	Protein binding	*Fndc3b*	Down	Mouse, human	[93]
Cdr1as	MI	miRNA sponge	*Cdr1*	Up	Mouse	[80]
circNCX1	Myocardial ischemia–reperfusion	miRNA sponge	*Ncx1*	Up	Mouse	[81]
circ-TTC3	MI	miRNA sponge	*Ttc3*	Up	Rat	[83]
circNfix	MI	miRNA sponge/protein binding	*Nfix*	Up	Human, rat, mouse	[84]
circHipk3	MI	miRNA sponge/protein binding	*Hipk3*	Down	Mouse	[85]
circCDYL	MI	miRNA sponge	*Cdyl*	Down	Mouse	[86]
MFACR	Myocardial ischemia–reperfusion	miRNA sponge	*Smyd4*	Up	Mouse	[87]
circRbms1	Myocardial ischemia–reperfusion	miRNA sponge	*Rbms1*	Up	Mouse	[88]
circSamd4	MI	Protein binding	*Samd4*	Down	Mouse	[89]
circ-ZNF609	Myocardial ischemia–reperfusion	Protein binding	*Znf609*	Up	Mouse, human	[92]
circ-SNRK	MI	miRNA sponge/protein binding	*Snrk*	Down	Mouse	[94]
circNFIB	MI	miRNA sponge	*Nfib*	Down	Mouse	[95]
circHIPK3	AngII-II-induced cardiac fibrosis and hypertrophy	miRNA sponge	*Hipk3*	Up	Mouse	[96]
circ_000203	AngII-II-induced cardiac fibrosis	miRNA sponge	*Myo9a*	Up	Mouse	[97]
circRNA_010567	Diabetic-induced cardiac fibrosis	miRNA sponge	*Zswim6*	Up	Mouse	[98]
circ_0036176	Myocardial fibrosis	miRNA sponge, protein coding	*Myo9a*	Up	Human, mouse	[99]
circANRIL	Atherosclerosis	Protein binding	*Ink4*	Down	Human	[102]
circCHFR	Atherosclerosis	miRNA sponge	*Chfr*	Up	Human	[104]
circ_Lrp6	Atherosclerosis	miRNA sponge	*Lrp6*	No change	Mouse	[105]
circNRG-1	AngII-induced vascular remodeling	miRNA sponge	*Ngr1*	Down	Mouse	[106]
circEsyt2	Atherosclerosis	Protein binding	*Esyt2*	Up	Mouse	[107]
cZNF292	Impaired endothelial cell function	Protein binding	*Znf292*	N/A	Human, mouse	[51]
cTTN1, cTTN2, cTTN3, cTTN4, cTTN5	Dilated cardiomyopathy	N/A	*Ttn*	Down	Human, mouse	[19]
cCAMK2D	Hypertrophic cardiomyopathy	N/A	*Camk2d*	Down	Human, mouse	[19]
circ-Amotl1	Doxorubicin-induced cardiomyopathy	Protein binding	*Amotl1*	Down	Human, mouse	[108]
circ-Foxo3	Doxorubicin-induced cardiomyopathy	Protein binding	*Foxo3*	Up	Mouse	[109]
circ-INSR	Doxorubicin-induced cardiomyopathy	Protein binding	*Insr*	Down	Human, mouse	[111]
CACR	Diabetic cardiomyopathy	miRNA sponge	*Slc29a1*	Up	Human	[112]
circBPTF	Diabetic cardiomyopathy	miRNA sponge	*Bptf*	Up	Human	[113]
CDR1as	Diabetic cardiomyopathy	miRNA sponge	*Cdr1*	Up	Mouse	[115]
circHIPK3	Diabetic cardiomyopathy	miRNA sponge	*Hipk3*	Down	Human	[116]
circSlc8a1	Pressure-overload-induced cardiac hypertrophy	miRNA sponge	*Slc8a1*	Up	Human, mouse	[118]
HRCR	Isoproterenol-induced cardiac hypertrophy	miRNA sponge	*Pwwp2*	Down	Mouse	[119]
circNlgn	Pressure-overload-induced cardiac hypertrophy	Protein encoding	*Nlgn*	Up	Human, mouse	[121]

### The role of circRNA in MI

The morbidity and mortality of MI remain high, despite the development of interventional coronary reperfusion approaches. Heart ischemia is associated with cardiomyocyte loss, a major contributing factor to cardiac dysfunction. Therefore, inhibiting cardiomyocyte apoptosis and promoting its survival are potential therapeutic approaches. To this end, the clinical significance of circRNA and its relationship with MI pathophysiology have been investigated in several studies. In one such study, Cdr1as overexpression was shown to promote mouse cardiac myocyte apoptosis and to exacerbate cardiac infarct size by targeting miR-7a [80]. circNCX1 suppression in mice’ hearts can reduce cardiac ischemia–reperfusion-induced cardiac injury by sponging miR-133a-3p, which would otherwise promote cardiomyocyte apoptosis [81,82]. In the rat myocardium, circ-TTC3, one of the abundantly expressed circRNAs, is significantly upregulated 5 weeks following MI [83]. In vitro and in vivo investigations suggest that circ-TTC3 inhibition aggravates MI-induced cardiac dysfunction, while circ-TTC3 overexpression prevents ischemia-stimulated cardiomyocyte apoptosis via circ-TTC3/miR-15b/Arl2 axis [83]. A super-enhancer is a cluster of enhancers that drive the expression of genes by recruiting transcription factors and by altering the epigenetic landscape. circNfix is a super-enhancer that is controlled by transcription factor Meis1. Inhibition of circNfix leads to the improvement of MI-induced cardiac dysfunction, as well as an increase in cardiomyocyte survival and angiogenesis. Mechanistically, circNfix acts dually. First, through ubiquitination, circNfix inhibits the expression of cyclin A2 and cyclin B1 by enhancing the interaction between Ybx1 and Nedd4l. On the other hand, circNfix serves as a sponge for miR-214 to regulate GSK3β expression and suppress β-catenin activity [84]. circHipk3 overexpression improves post-MI-induced cardiac dysfunction and attenuated cardiac fibrosis via inhibiting N1ICD degradation and sponging miR-133a [85]. Overexpression of circCDYL can promote cardiac repair after acute MI through sponging miR-4793-5p [86]. circRNA MFACR (mitochondrial fission and apoptosis-related circRNA) modulates the expression of MTP-18 through sponging miR-652-3p [87]. Knockdown of MTP-18, a mitochondria fission-related mitochondrial membrane protein, significantly reduces the fragmented mitochondria and suppresses cardiomyocyte apoptosis [87]. Finally, the circRNA circRbms1 has also shown substantial potential to regulate myocardial ischemia injury through its inhibition, which alleviates myocardial apoptosis and reactive oxygen species production [88]. In addition to act as miRNA sponges, circRNAs can also interact with proteins and participate in MI pathophysiology. For example, mitochondria-localized circRNA circSamd4 overexpression promotes cardiomyocyte proliferation, prevents cardiomyocyte apoptosis, and consequently ameliorates MI-induced cardiac dysfunction by interacting with VCP protein and modulating the mitochondrial permeability transition pore [89]. circ-ZNF609 is a ubiquitously expressed circRNA, it widely participates in the regulation of myogenesis, cancer cell migration, and growth by sponging miRNAs as well as to be translated into protein itself [90,91]. In the heart, circ-ZNF609 inhibition prevents heart ischemia/reperfusion injury [92]. In cardiomyocyte, circ-ZNF609 directly binds to YTHDF3 protein, and fine-tunes the accessibility of *Yap* mRNA to YTHDF1 and YTHDF2, consequently, regulating the expression of YAP. Overexpression of circ-ZNF609 aggravates cardiomyocyte apoptosis and disrupts the cross-talk between Hippo-YAP and Akt signaling via downregulating YAP expression [92]. The circRNA circFndc3b is downregulated in mice’ hearts following MI. Furthermore, the human ortholog of circFndc3b is also significantly downregulated in the cardiac tissues of patients with ischemic cardiomyopathy. Overexpression of circFndc3b improved cardiac function by reducing cardiomyocytes apoptosis via modulating FUS/vascular endothelial growth factor signaling [93]. circ-SNRK overexpression improves the cardiac function after MI in rats. Interestingly, circ-SNRK was found to maintain the proper protein level of SNRK by a negative feedback mechanism and, thus, regulate cardiomyocyte energy metabolism [94]. Collectively, these findings suggest that the occurrence and progression of MI are highly associated with circRNAs expression and regulation. Further investigation to understanding the underlying mechanism of circRNA and its therapeutic application in MI are warranted.

### The role of circRNA in cardiac fibrosis

The severity of cardiac fibrosis correlates with higher long-term mortality in patients with cardiac disease, especially heart failure. Cardiomyocytes, fibroblasts, and vascular cells in the heart are connected by a complex matrix, which helps to maintain the cardiac plasticity and structural integrity. When the heart is compromised, the balance between fibroblasts and cardiomyocytes is disrupted. An increase in collagen synthesis or a decrease in collagen degradation results in an excessive, diffuse accumulation of collagen in the interstitial and perivascular tissues. circRNA circNFIB is downregulated both in the heart after MI and in primary adult cardiac fibroblasts when treated with transforming growth factor-β. Conversely, overexpression of circNFIB inhibits primary fibroblast proliferation via sponging miR-433 [95]. circHIPK3 is also significantly elevated in cardiac fibroblasts treated with angiotensin II (AngII), and its suppression alleviates AngII-stimulated cardiac fibrosis [96]. Mechanism-wise, circHIPK3 functions as a miR-29b-3p sponge and therefore regulates the fibrotic-associated target genes (α-smooth muscle actin, COL1A1, and COL3A1) of miR-29b-3p [96]. Similarly, circ_000203 upregulates the fibrotic genes (COL1A2, COL3A1, and α-smooth muscle actin) by interacting with miR-26b-5p [97]. In a diabetic mouse model, the expression level of circRNA_010567 in the heart is increased, and circRNA_010567 knockdown inhibits the secretion of fibrosis-related proteins in cardiac fibroblasts through circRNA_010567/miR-141/transforming growth factor-β1 [98]. Aside from acting as a miRNA sponge, circRNA can also encode proteins that exert regulatory effects on cardiac fibroblasts. For instance, circRNA circ_0036176 can encode a 208-amino-acid protein (Myo9a-208) that suppresses cardiac fibroblast proliferation. Interestingly, miRNA miR-218-5p can directly bind to circ_0036176 and inhibit the translation of Myo9a-208 [99]. Cardiac fibrosis is caused by the accumulation of extracellular collagen proteins in the heart leading to systolic or diastolic dysfunction. In advanced stages, fibrosis eventually leads to severe organ dysfunction and death. Detection, prevention, and reversal of cardiac fibrosis are important strategies for the treatment of heart failure [100]. Currently, while most studies on how circRNAs regulate cardiac fibroblasts are focused on circRNA/miRNA-mediated regulation of fibrotic-associated genes expression, other regulatory functions of circRNA in cardiac fibrosis should be further explored.

### The role of circRNA in atherosclerosis

It is now well established that atherosclerosis, endothelial cell dysfunction, vascular smooth muscle cell (VSMC) alteration, excess lipid, cholesterol accumulation, and inflammation are critical for the development of atherosclerosis [101]. The occurrence of atherosclerosis generally involves the deposition of lipids and other blood components in the arterial intima, the proliferation of smooth muscle cells, and the increase of collagen fibers. Thus, reducing the lipid deposition and inhibiting smooth muscle cell proliferation can effectively slow down the pathological process of atherosclerosis. circANRIL, which is located at the CVD risk locus on chromosome 9p21, can induce cell apoptosis and inhibit proliferation via binding to PES1 to prevent rRNA maturation [102]. circANRIL elevation could activate p53 and exert atheroprotective effects, indicating its potential to be used as therapeutic target for atherosclerosis treatment [102]. Using RNA sequence, the circRNA, miRNA, and mRNA expression were profiled in rabbits with high-fat diet-induced atherosclerosis or normal diet. Dysregulated circRNA-associated competitive endogenous RNA network in atherosclerosis was analyzed and identified 7 circRNAs as potential diagnostic factors for atherosclerosis [103]. circRNA circCHFR was found to be upregulated in the oxidized low-density lipoprotein-induced VSMCs, and its suppression was found to inhibit the proliferation and migration of VSMCs through sponging miR-370 and its target gene FOXO1 [104]. Another circRNA circ_Lrp6 when suppressed can significantly reduce the VSMC migration and proliferation. Another study demonstrates that circ_Lrp6 functioned as a native sponge of miR-145 and consequently regulated the miR-145’s targets *Fascin*, *Lox*, and *Yes1* [105]. circNRG-1 is identified as an important circRNA regulator in modulating AngII treatment-induced VSMC anti-apoptotic effects [106]. Mechanistically, circNRG-1 promotes NRG-1 degradation by sponging miR-193b-5p [106]. Overexpression of circEsyt2 promotes VSMC proliferation and migration and suppresses VSMC apoptosis by directly binding to PCBP1 and facilitating p53β splicing [107]. cZNF292 knockout regulates the endothelial cell flow responses and leads to abnormal endothelial morphology SDOS specifically binds to cZNF292, and any SDOS mutations that disrupt the interaction between SDOS and cZNF292 can also abolish the laminar flow response [51]. Nonetheless, despite reports of studies investigating circRNAs’ function in atherosclerosis, the underlying role of circRNA has not yet been fully elucidated, and further research is necessary.

### The role of circRNA in cardiomyopathy

Cardiomyopathy is the progressive cardiac insufficiency caused by structural changes in the ventricle that ultimately impair cardiac function. Here, we will discuss mainly 3 types of cardiomyopathies: dilated cardiomyopathy, hypertrophic cardiomyopathy, and diabetic cardiomyopathy. Dilated cardiomyopathy is often characterized by an enlarged cardiac chamber and accompanied by systolic myocardial dysfunction, while hypertrophic cardiomyopathy is caused by genetic mutations and has a similar phenotype to pressure overload-induced cardiac hypertrophy. Diabetic cardiomyopathy is a diabetes-related cardiovascular complication that is accompanied by cardiomyocyte metabolic disorder, myocardial interstitial fibrosis, and cardiac dysfunction. A study that performed ribosomal depleted RNA sequencing from human hearts and RBM20-null mice found 6 circRNAs (cTTN1, cTTN2, cTTN3, cTTN4, cTTN5, and cCAMK2D) that were significantly downregulated in response to dilated cardiomyopathy, while only one circRNA (cCAMK2D) was decreased in the heart with hypertrophic cardiomyopathy [19]. In doxorubicin-induced cardiomyopathy, a condition usually manifested as dilated cardiomyopathy, circ-Amotl1 overexpression was shown to confer positive therapeutic effects. In primary cardiomyocytes, circ-Amotl1 directly interacts with PDK1 and AKT1 to facilitate the nuclear translocation of phosphorylated AKT [108]. circRNA circ-Foxo3 has been found to suppress cell proliferation by forming cyclin-dependent kinase 2/circ-Foxo3/p21 ternary complex [109]. Interestingly, forced expression of circ-Foxo3 can exacerbate doxorubicin-induced cardiomyopathy via interacting with antisenescent proteins ID-1 and E2F1 [110]. The number of different regulatory mechanisms of circ-Foxo3 indicates that the stress response pathways of circRNAs may be different each time depending on the stressors. Recently, circ-INSR has been found to bind to SSBP1 protein and exert cardioprotective effects on doxorubicin-induced cardiomyopathy [111]. In vitro cyclized circ-INSR can prevent cardiomyocyte doxorubicin-induced cell death [111]. In addition to doxorubicin-induced ventricular dilatation, circRNAs also participate in the regulation of diabetic cardiomyopathy. circRNA CACR silencing could alleviate high-glucose-induced cardiomyocyte pyroptosis [112]. It is worth noting that the expression level of CACR in diabetic patients’ serum was increased, indicating the potential diagnostic value of CACR for diabetic cardiomyopathy. Besides, circBPTF and circ_0071269 knockdown and CDR1as and circHIPK3 overexpression can attenuate diabetic cardiomyopathy [113–117]. Currently, those studies indicate that pathology of cardiomyopathy could be mediated by circRNAs; however, future studies are needed to fully elucidate the clinical significance and the mechanisms of circRNA on cardiomyopathy.

### The role of circRNA in cardiac hypertrophy

Pathological cardiac hypertrophy in patients with MI, valvular disease, and metabolic syndrome is initially characterized by reduced ventricular size and increased wall thickness, leading to ventricular dilatation, and impaired systolic function (maladaptive remodeling), eventually resulting in heart failure. circRNAs are implicated in cardiac hypertrophy via several pathways. Knocking down the highly abundant circRNA *circSlc8a1* suppresses pressure-overload-induced cardiac hypertrophy via endogenously sponging miR-133 [118]. circRNA HRCR acts as an endogenous sponge of miR-223, expression of which can exacerbate cardiac hypertrophy, whereas HRCR elevation inhibits isoproterenol-induced cardiac hypertrophy [119]. Harnessing the sponging activity of circRNA, an engineered circRNA circmiR that acts as miR-132 and miR-212 sponge was shown to be effective in attenuating transverse-aortic-constriction-induced cardiac hypertrophy [120]. Another circRNA circNlgn can encode a peptide named Nlgn173. Aberrant expression of Nlgn173 results in cardiac fibroblast proliferation and aggravates pressure-overload-induced cardiac hypertrophy [121]. In summary, a relatively small number of studies have examined the role of circRNAs in cardiac hypertrophy. However, whether other circRNAs also play a role in regulating cardiac hypertrophy or the underlying mechanisms remains unclear, and further research is needed.

## circRNAs as Biomarkers for CVDs

circRNAs are abundant in blood, urine, and extracellular vesicles and exhibit high stability and differential expression in response to stress stimulus conditions [122]. Emerging evidence indicates that circRNAs can be used as diagnostic and prognostic biomarkers for CVDs (Table 4). Serum circR-284:miR-221 ratio is significantly associated with the carotid-related cerebrovascular ischemic events and, thus, may act as potential diagnostic biomarker for carotid-related ischemic stroke [123]. circRNA MICRA (also named circ-ZNF609) is significantly decreased in the blood of patients with MI and acts as a prognostic biomarker of left ventricular dysfunction after MI [124,125]. Through performing circRNA microarray in peripheral blood mononuclear cells of patients with coronary artery disease, hsa_circ_0001879 and has_circ_0004104 are identified as diagnostic biomarkers for coronary artery disease [126]. Similarly, peripheral has_circ_0124644 (in peripheral blood), hsa_circ_0001445 (in plasma), and hsa_circ_0005540 (in extracellular vesicles) are validated as biomarkers for coronary artery disease with high diagnostic value [127–129]. Plasma hsa_circ_0062960 in patients with heart failure is significantly increased compared with normal groups, and a high association between the expression of hsa_circ_0062960 and serum BNP has been displayed, exhibiting great potential of hsa_circ_0062960 to be utilized as a diagnostic biomarker in patients with heart failure [130]. Another candidate, hsa_circ_0097435, is also upregulated in the peripheral blood of patients with heart failure [131]. In a similar study, plasma circRNAs are evaluated in children with chronic heart failure. Three circRNAs (has_circRNA_004183, has_circRNA_079265, and has_circRNA_105039) were found downregulated, and receiver operating characteristic curve analysis indicated that the combination of the 3 circRNAs could effectively be used as diagnostic biomarker for the development of chronic heart failure in children [132]. In addition, serum circRNAs in patients with hypertrophic cardiomyopathy were investigated, and 3 (circDNAJC6, circTMEM56, and circMBOAT2) were found to be downregulated, among which circTMEM56 and circDNAJC6 had a negative correlation with hypertrophic obstructive cardiomyopathy outcome [133]. Interestingly, it is worth noting that endurance training also alters the plasma circMBOAT2 levels, demonstrating that circRNAs can serve as a potential predictor for disease prognosis and cardiopulmonary adaptation [134]. To conclude, it is anticipated that more circRNAs will be identified as biomarkers for CVD as circRNA exploration progresses, contributing to accurate diagnosis and prognosis.

**Table 4.** circRNA as biomarkers for CVD.

**circRNAs**	**Biofluids**	**Disease**	**Application**	**References**
circR-284	Serum	Carotid-related ischemic stroke	Diagnostic biomarker	[123]
MICRA (also named circ-ZNF609)	Peripheral blood	Patients with MI	Prognostic biomarker	[124,125]
hsa_circ_0001879, has_circ_0004104	Peripheral blood	Coronary artery disease	Diagnostic biomarker	[126]
has_circ_0124644	Peripheral blood	Coronary artery disease	Diagnostic biomarker	[127]
hsa_circ_0001445	Plasma	Coronary artery disease	Diagnostic biomarker	[128]
hsa_circ_0005540	Plasma	Coronary artery disease	Diagnostic biomarker	[129]
hsa_circ_0062960	Plasma	Patients with heart failure	Diagnostic biomarker	[130]
hsa_circ_0097435	Peripheral blood	Patients with heart failure	Diagnostic biomarker	[131]
has_circRNA_004183, has_circRNA_079265, has_circRNA_105039	Plasma	Children with chronic heart failure	Diagnostic biomarker	[132]
circTMEM56, circDNAJC6, circMBOAT2	Serum	Patients with hypertrophic cardiomyopathy	Diagnostic biomarker	[133]
circMBOAT2	Plasma	Endurance training	Prognostic cardiopulmonary adaption	[134]

## circRNA-Based Therapeutic Applications in CVDs

RNA-based therapies have emerged to be promising treatment strategies for many diseases and vaccine development [58,135]. One of the major challenges in developing circRNA-based therapeutic strategies that target endogenous circRNAs of interest, similar to gain- and loss-of-function studies that we have discussed in methodologies for circRNA functional determination, is to generate circRNA overexpression and inhibition constructs efficiently without affecting their linear counterparts. Besides, artificial circRNAs, which are designed as miRNA sponges or encode proteins, are also attractive application strategies and exhibit promise in disease therapy [59]. For instance, an artificial circRNA (circmiR) generated to act as miR-132/-212 sponge can prevent pressure-overload-induced cardiac hypertrophy [120]. Although the circRNA-based therapeutic strategy is promising, many challenges remain.

**Fig. 6.**
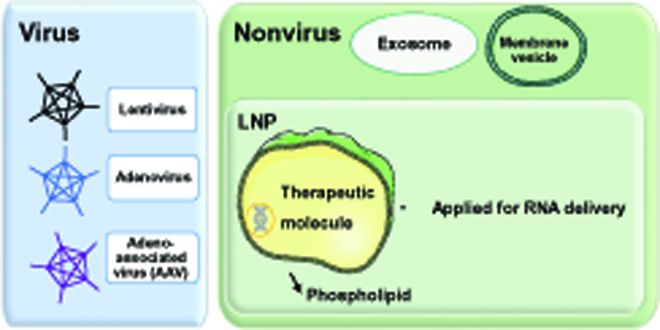
Flowchart of circRNA therapeutic strategies based on different delivery systems. Lentivirus, adenovirus, and AAVs are the most frequently used virus systems for RNA delivery, while exosomes (and other cell-derived membrane vesicles) and LNPs are the common nonvirus carriers for RNA delivery.

Efficient delivery of RNA into cells and organs is a major challenge that needs to be addressed before RNA therapy can be applied clinically. RNA encapsulation mediated by viral or nonviral vectors represents the most common delivery strategy (Fig. 6). Several virus-based delivery systems are developed and applied to RNA-based therapy. Lentivirus, adenovirus, and adeno-associated virus (AAVs) are the most frequently used virus systems for RNA delivery. Among them, the AAV system is one of the most promising viral delivery systems in translational medical research due to its low immunogenicity. Unlike lentiviruses, AAVs do not integrate DNA into the host cells’ genome, and, thus, unwanted off-target gene expression changes are avoided. Currently, most animal studies that study the cardiovascular system are focused on circRNA endogenous interference via AAV delivery systems. Despite the advantages of the AAV delivery system, limitations are still present, including the presence of a large population of neutralizing antibodies against AAVs. Future design and development of clinically applicable AAV vectors to improve the efficacy and safety of human gene therapy remain essential and urgent.

To deliver circRNAs in sufficient amount and in a safe manner, nonvirus delivery systems are also developed. Exosome and other cell-derived membrane vesicles and lipid nanoparticles (LNPs) are considered common carriers for RNA delivery [136]. LNPs are important drug delivery systems approved by the Food and Drug Administration for use in small RNA and mRNA delivery [137]. For example, nanoparticle delivery of circ-Amotl1 plasmids is effective in attenuating doxorubicin-induced cardiotoxicity [108]. However, this approach is not without side effects, as it has a relatively low proportion of circularized RNA to linear RNAs and inadvertently activates the innate immune system. In another mRNA-based approach, used for therapy application, in vitro transcribed mRNA with capped and poly(A) structures directly targeting the myocardium are applied as an effective therapeutic approach to cardiac disease treatment. For example, purified vascular endothelial growth factor mRNA was delivered into cardiomyocytes and improved the cardiac function in MI swine [138]. Moreover, LNPs delivered modified mRNA encoding CAR to T lymphocytes to improve cardiac function and prevent cardiac injury [139]. Currently, studies about in-vitro-synthesized circRNA-based therapeutic studies are still in infancy. Recently, circRNA vaccines that induce effective neutralizing antibody production and T cell immune response to protect against severe acute respiratory syndrome coronavirus 2 have been developed and delivered via LNPs [58]. In the cardiovascular systems, in-vitro-synthesized circRNA circ-INSR directly transfected into cultured neonatal rat cardiomyocytes alleviated doxorubicin-induced cardiomyocyte injury [111]. circmiR, in vitro synthesized by a group I PIE, demonstrates greater molecule stability than its linear counterpart [120]. However, in-vitro-synthesized circRNAs still need to overcome technical hurdles, such as extraneous fragments or imperfect cyclization that can lead to immune response activation. In addition to the improvement of delivery systems, extending RNA half-life, reducing immune response, increasing protein-coding efficiency, and inhibiting off-target effects also need to be optimized in developing circRNA-based drugs.

## Conclusions and Future Perspectives

In this review article, we review the research progress and therapeutic applications of circRNAs in CVDs. circRNAs are involved in variety of cellular processes and are associated with many human diseases. In recent years, new technologies and methods for circRNA identification, validation, functional study, and therapy applications have been developed. In the cardiovascular system, however, the underlying regulatory mechanisms of circRNAs are still to be explored in depth. Most studies have focused on the function of circRNAs as miRNA sponge, while other functions, mechanisms, and pathways have been less studied. There is still a need for further in-depth mechanistic research to be carried out. Besides, given the increasing morbidity and mortality of CVDs, the need for development of new diagnostic and prognostic biomarkers is critical. The potential of circRNAs for biomarker development is enormous, as these molecules exhibit excellent stability in biofluids, evolutionary conservation, and various distinctive functional aspects. However, our knowledge on the biology of circRNAs is currently limited, making the application of circRNAs in clinical practice very challenging. Hence, further in-depth functional and structural studies to better understand the biology of circRNAs are required before these new therapeutic regimens can be routinely implemented.

The aim of this review is to differentiate from previously summarized reviews regarding this topic by focusing on the research progress and challenges that have been faced in the development of circRNA-based therapy for the prevention of CVDs. With the advancement of RNA biology and RNA-related technologies, RNA-based drug design and drug delivery systems have achieved more diversified development, and RNA therapy has entered a new era of rapid development [135]. Because of its unique circular characteristics, circRNA has shown great application potential in the fields of innovative drug (synthesized circRNA with endogenous sequences and engineer-designed artificial circRNA) and vaccine development compared with traditional RNA therapy. Many reviews in the literature have summarized the research progress on circRNA in cardiovascular system in the past few years, but with rapid advancements in circRNA study, our knowledge of circRNA and its application in medical research is constantly evolving [11,140–143]. In this review, we summarize the most popular databases and methods in cardiovascular research about circRNAs and provide the latest information about circRNA biogenesis and regulation. In addition, we discuss the latest techniques that have been or may be used to study circRNAs in the cardiovascular system, as well as their advantages and disadvantages. Hopefully, this information will be of use to junior investigators as guidelines for conducting circRNA research in the cardiovascular system and promote the advancement of this field. Overall, throughout this review, we address the current state-of-the-art knowledge of circRNAs, as well as therapeutic strategies, application prospects, and challenges associated with circRNAs in CVDs.

The growing understanding of the different roles of RNAs has led to the development of new RNA-based drugs. RNA therapy has the potential to be a powerful new therapeutic strategy to address previously untreatable diseases. One of the most attractive areas of circRNA research is the circRNA-based drug design for CVD therapy. With the progressive understanding of the role of circRNAs in CVDs and circRNA-mediated RNA therapy, a new way is paved for CVD diagnosis and treatment. However, there are still barriers to overcome. Many efforts have been made to synthesize circRNAs effectively. Currently, most circRNAs are generated directly in vivo by overexpressing strategies. However, these strategies harbor the danger of coproducing corresponding linear RNA as side product that can compromise the quality and purity of the newly produced circRNAs. To circumvent this problem, synthesized circRNA must be directly delivered into the target similarly to mRNA therapy. Moreover, the application of engineered circRNA synthesis strategies is usually limited because of low cyclization efficiency and excessive immune response stimulation caused by artificially introduced exogenous fragments. The development of improved circRNA production methodologies will enable further important research on the open questions about circRNA function and allow for the development of new circRNA-based technologies. The circRNA mode of delivery is another important challenge that needs to be overcome. For example, as mentioned earlier, nanoparticles can either inadvertently activate the immune system or sometimes be accumulated into other unwanted tissues causing side effects. Therefore, further research focusing on reducing the side effects of nanoparticles and improving their safety profile is essential to the clinical application of circRNA drugs.

Vaccine development is another promising application of circRNA research. Although mRNA-based vaccines have now established themselves as a component of immunotherapy, progress in the clinical development of mRNA vaccines has been hampered by molecule instability and storage problems. Nonetheless, mRNA-based vaccines performed well during the coronavirus disease 2019 pandemic in preventing coronavirus disease 2019 variants [144]. Because of the rapid progress in coronavirus disease vaccine development and implementation, many mRNA vaccines are currently in clinical trials for various diseases [145,146]. The advantage of circRNA over other RNA vaccine technologies is that it utilizes cap-independent translation that confers a highly stable structure, as no nucleotide modification is needed for its activity. More importantly, however, the application of circRNA-based vaccine development and implementation in CVDs is still largely unexplored. Thus, it is only logical that capitalizing on the great potential of circRNA to develop therapeutic (drug) and preventive (vaccine) modalities for CVD can be a viable and groundbreaking future step to take. In order for this to be realized, important challenges of circRNA-based applications, including optimized circRNA synthesis strategies and better delivery systems, must be overcome.

## Acknowledgments

**Funding:** This work was supported by the grants from National Key Research and Development Project (2018YFE0113500 to J.X.), National Natural Science Foundation of China (82020108002 and 82225005 to J.X. and 82270291 to L.W.), Innovation Program of Shanghai Municipal Education Commission (2017-01-07-00-09-E00042 to J.X.), the grant from Science and Technology Commission of Shanghai Municipality (21XD1421300 and 20DZ2255400 to J.X.), the “Dawn” Program of Shanghai Education Commission (19SG34 to J.X.), and ZonMw PSIDER grant (no. 10250022110004) to J.P.G.S. **Competing interests:** The authors declare that they have no competing interests.
